# Enhancing Pediatric Emergency Medicine Skills Through Entrustable Professional Activity-Based Simulation for Third-Year Medical Students

**DOI:** 10.7759/cureus.110865

**Published:** 2026-06-15

**Authors:** Marc Berenson, Kyrillos E Attaalla, Christin Traba, Kei U Wong

**Affiliations:** 1 Emergency Medicine, Stanford University School of Medicine, Stanford, USA; 2 Emergency Medicine, Rutgers New Jersey Medical School, Newark, USA; 3 Pediatrics, Rutgers New Jersey Medical School, Newark, USA

**Keywords:** curriculum development, entrustable professional activities, neonatal resuscitation, pediatric emergency medicine, simulation-based education, undergraduate medical education

## Abstract

Background

The pediatric clerkship rotation exposes medical students to essential skills for managing a wide range of pediatric conditions, including emergency care. Simulation-based education is a mainstay of medical education that provides an effective means of developing clinical decision-making skills, especially in high-risk situations. The addition of simulation education to pediatric clerkships would provide direct observation of Core Entrustable Professional Activities (EPAs), competencies defined by the Association of American Medical Colleges as "the abilities that new physicians should be able to demonstrate upon the first day of internship".

Objective

The study aims to evaluate the effectiveness of an EPA-based simulation education program in a pediatric clerkship that focuses on neonatal resuscitation, informed consent, and lumbar puncture.

Methods

A structured simulation education program was incorporated in a pediatric clerkship that utilized case-based scenarios, partnered informed consent education sessions, and procedure skill trainers. A pre- and delayed post-assessment was used to evaluate changes in self-efficacy and knowledge retention. Feedback was used to assess the perceived educational benefits.

Results

The study showed that students' self-efficacy in managing pediatric emergencies has significantly improved. Objective measurements showed that knowledge retention improved significantly after six weeks.

Conclusion

The integration of simulation-based education into pediatric clerkship effectively enhances medical students' preparedness to manage pediatric emergencies. The addition of EPA-based simulation education to pediatric clerkships would better prepare medical students to manage pediatric emergencies.

## Introduction

The Association of American Medical Colleges (AAMC) published the "Core Entrustable Professional Activities (EPAs) for Entering Residency" in 2014, a guide designed to help transform US medical schools' assessment paradigms into a competency-based format in which students are evaluated based on directly observable behaviors required for entry into postgraduate training programs [1]. While direct observation is ideal, there are limitations to medical students' ability to demonstrate competence in specific skill sets due to a lack of patient consent, limited opportunities in high-acuity or low-frequency clinical encounters, or the inappropriateness of performing specific actions at their current training stage [2-5]. Furthermore, previous studies found that medical students received inadequate supervision during clinical tasks, limiting the success of this framework [6]. Difficult-to-observe AAMC EPAs, including EPA 10: recognizing a patient requiring urgent care and initiating evaluation and management; EPA 11: obtaining informed consent for procedures; and EPA 12: performing general procedures, highlight the need for innovative, feasible approaches to ensuring learner proficiency and competence in these areas [1].

Historically, medical schools have relied on Objective Standardized Clinical Examinations to verify critical clinical skills before certifying students as ready for graduation. However, these clinical encounters are limited to standardized patients who are generally not critically ill and typically do not require urgent procedures. Simulation-based education has proven effective in teaching critical care skills, resulting in the long-term retention of knowledge and procedural abilities [7-9]. In one study, more than 98% of respondents reported an increase in theoretical knowledge and practical skills; in another study, time to transfusion in real-life patients with postpartum hemorrhage was reduced by 50% after a simulation [10,11]. As such, simulation-based education offers a unique opportunity to help students acquire the necessary skills outlined in the EPAs for postgraduate training. Simulation-based education is also effective for teaching pediatric emergency medicine content. Ansquer et al. noted satisfaction and significant gains in knowledge, skills, and clinical practice, particularly in anticipation, procedural skills, and teamwork [12]. They supported integrating such training into pediatric emergency medicine curricula.

Utilizing a competency-based framework, a novel three-part simulation-based curriculum was developed and implemented in the pediatrics core clerkship, aligned with AAMC EPAs 10, 11, and 12. These EPAs were selected because they represent clinically important skills that are essential for entering residency but may be difficult for medical students to perform or be directly observed doing in the clinical environment. The curriculum consisted of a three-hour session with three distinct exercises: (1) EPA 10: a team-based simulation case focused on the management of neonatal sepsis with hypoglycemia; (2) EPA 11: peer-to-peer informed consent practice for lumbar puncture; and (3) EPA 12: performance of lumbar puncture [1]. To our knowledge, no study has been performed on a simulation curriculum aligned with multiple EPAs for use in a pediatric core clerkship. This study aimed to assess the effect of the curriculum on learner self-efficacy and knowledge acquisition/retention as primary outcomes, with experiential learning and perceived simulation impact assessed as secondary outcomes. We hypothesized that this EPA-based simulation curriculum would improve students' self-efficacy and knowledge related to neonatal resuscitation, informed consent, and lumbar puncture.

## Materials and methods

This was a single-center, quantitative analysis of pre- and post-survey data after a simulation-based educational experience targeting AAMC's EPAs 10, 11, and 12. The study was conducted at Rutgers New Jersey Medical School in Newark, New Jersey, from May 2022 to March 2023 after obtaining approval from the Rutgers Institutional Review Board Committee (approval number: Pro2022002349). Participants were eligible for inclusion if they were third-year medical students completing their mandatory pediatrics core clerkship at a US medical school during the study period. There were no exclusion criteria. No formal sample size calculation was performed, as the study included a census of a predefined cohort of third-year medical students enrolled in the pediatric clerkship during the study period. 

A survey instrument was administered immediately prior to the simulation, which occurred during the first week of the pediatric clerkship (Appendix A). After the six-week clerkship, the survey instrument was repeated with additional questions regarding students' clinical experience with these tasks during their clerkship and their perceptions of how the simulation influenced their clinical performance during their pediatric rotation (Appendix B). EPA 10 was assessed through a team-based simulation case in which students had to recognize and initiate management of a critically ill three-week-old infant presenting with fever, hypoglycemia, and concern for neonatal sepsis. During the case, students were expected to evaluate the neonate for instability, obtain relevant history, initiate resuscitative management, develop a differential diagnosis, and create a plan that included a lumbar puncture. EPA 11 was assessed through a peer-to-peer informed consent exercise for lumbar puncture, during which students practiced explaining the indication, risks, benefits, alternatives, and steps of the lumbar puncture. EPA 12 was assessed through hands-on practice of lumbar puncture using a task trainer under faculty supervision. 

The survey instrument consisted of 10 multiple-choice knowledge questions assessing recognition and management of neonatal sepsis, obtaining informed consent, and performing a neonatal lumbar puncture. The survey also included experiential questions assessing students' prior clinical exposure to these tasks and five self-efficacy questions evaluating students' perceived ability to assess a critically ill pediatric patient, participate in the initial resuscitation of a critically ill infant, obtain informed consent, describe indications and contraindications for diagnostic lumbar puncture, and perform a diagnostic lumbar puncture. The study team developed the survey instrument to align with the curricular objectives and selected EPAs. Items were reviewed by faculty with expertise in pediatric emergency medicine, emergency medicine education, and simulation-based education for content alignment with the curriculum objectives. The instrument was not externally validated. 

Students completed the pre-survey before participating in the simulation curriculum. After the six-week clerkship, the survey instrument was repeated with additional questions regarding students' clinical experience with these tasks during the clerkship and their perceptions of how the simulation influenced their ability to engage in these clinical activities during their pediatric rotation. Only learners with matched pre- and post-survey responses were included in the paired analysis. Incomplete or unmatched responses were excluded from paired pre-post analysis. 

Data were collected using Qualtrics (Qualtrics, LLC, Provo, Utah, United States) and analyzed using Microsoft Excel (Microsoft Corporation, Redmond, Washington, United States). Given the ordinal nature of the Likert-scale data and the non-parametric distribution of responses, pre- and post-survey results were compared using the Wilcoxon signed-rank test. Knowledge assessment scores were compared before and after the curriculum to evaluate short-term knowledge retention at the conclusion of the clerkship. Because this was an exploratory educational study, no formal correction for multiple comparisons was applied. 

Logistics

Students were provided with pre-reading materials and instructional videos before the simulation session to ensure the simulation time was as effective and efficient as possible. A three-hour session was scheduled during the first week of the pediatrics clerkship, with a limit of 24 students per session. The session began with a 20-minute introduction covering objectives and expectations, followed by the collection of pre-survey responses. Students were then divided into small groups of 6-8 participants. The first small-group exercise was EPA 10: a faculty-facilitated, team-based, low-fidelity simulation scenario involving a three-week-old patient presenting to the pediatric emergency department with fever and ultimately diagnosed with neonatal sepsis and hypoglycemia. This exercise included a five-minute pre-briefing, a 20-minute small-group simulation, a 30-minute large-group debriefing, and a 20-minute didactic session. In the second part, students alternated between two 30-minute exercises: EPA 11: peer-to-peer practice in obtaining informed consent for lumbar puncture and EPA 12: performance of lumbar puncture on a task trainer in small groups under faculty supervision.

The three-hour simulation curriculum session concluded with a 15-minute summary followed by a question-and-answer (Q&A) session during the closing remarks. Post-session surveys were distributed at the end of week six of the clerkship to gather feedback.

## Results

In this study, 165 third-year medical students participated in a three-part EPA-based clinical skills simulation curriculum. Of these 165 students, 122 (73.9%) completed both pre- and post-surveys, allowing for paired data analysis. The survey assessed three domains: self-efficacy, experiential learning, and knowledge. Given the non-parametric nature of the data and its non-normal distribution, a Wilcoxon signed-rank test was used for the pre-post analysis.

Self-efficacy domain

**Figure 1:**
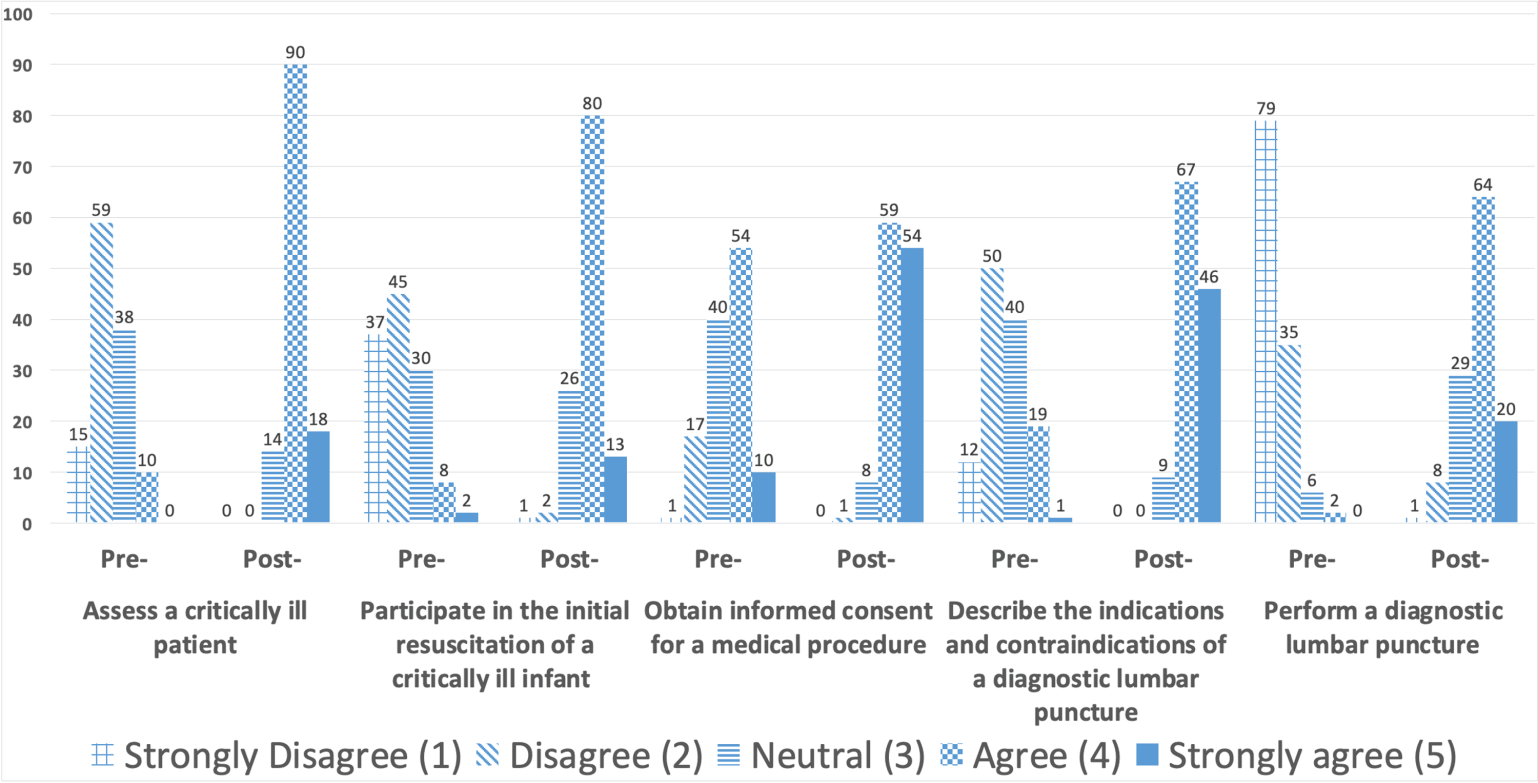
Pre- and post-intervention responses to the self-efficacy item: "I feel able to _____" Q1: Self-Efficacy Questions (Pre-Survey): Please respond to the following prompt: I feel able to _____. Self-efficacy was measured using a 5-point Likert scale (1=strongly disagree, 2=disagree, 3=neutral, 4=agree, 5=strongly agree), with scores compared before and after the educational intervention.

Among the 122 respondents who completed the post-curriculum survey after the six-week clerkship, significant improvements were observed across all five self-efficacy measures following the simulation curriculum (Figure 1). Self-efficacy was rated on a 5-point Likert scale: 1=strongly disagree, 2=disagree, 3=neutral, 4=agree, and 5=strongly agree. Table 1 presents the arithmetic mean of the median scores from respondents' self-efficacy ratings, assessed using a 5-point Likert scale, across pre- and post-survey data. The table summarizes the distribution of self-efficacy scores before and after the curriculum. Students reported increased confidence in assessing critically ill patients (p<0.01), with a median score of 4.03 out of 5 on a 5-point Likert scale, indicating general agreement with this statement. Specifically, 89% of respondents agreed or strongly agreed with the statement. Similarly, confidence in participating in the initial resuscitation of a critically ill infant also increased among the students (p<0.01), from a median score of 2.12 to 3.84. This shift demonstrates that students moved from a perceived lack of efficacy toward a more neutral but increasingly positive self-assessment of their abilities. Students perceived an improved ability to obtain informed consent for a medical procedure (p<0.01), with median scores increasing from 3.46 (reflecting neutrality) to 4.36 (indicating agreement), reflecting greater confidence in this aspect of medical practice. Regarding lumbar puncture, students' perception of their understanding of indications and contraindications also improved (p<0.01), with scores increasing from 2.57 (reflecting disagreement) to 4.30 (indicating agreement). Self-perceived preparedness to perform a lumbar puncture increased (p<0.01), with a median score rising from 1.43, indicating strong disagreement, to 3.77. This shift represents a move toward a more confident, neutral stance regarding students' ability to perform the procedure.

**Table 1:** Comparative analysis of pre- and post-survey median scores for the arithmetic means of median self-efficacy measures

Question	Average median score of question pre-simulation on a 5-point Likert scale	Average median score of question post-simulation on a 5-point Likert scale	P-value
Self-Efficacy Questions: Please respond to the following prompt: I feel able to _____.			
Assess a critically ill patient	2.35	4.03	<0.01
Participate in the initial resuscitation of a critically ill infant	2.12	3.84	<0.01
Obtain informed consent for a medical procedure	3.45	4.36	<0.01
Describe the indications and contraindications of diagnostic lumbar puncture	2.57	4.30	<0.01
Perform a diagnostic lumbar puncture	1.43	3.77	<0.01

Experiential learning domain

**Figure 2:**
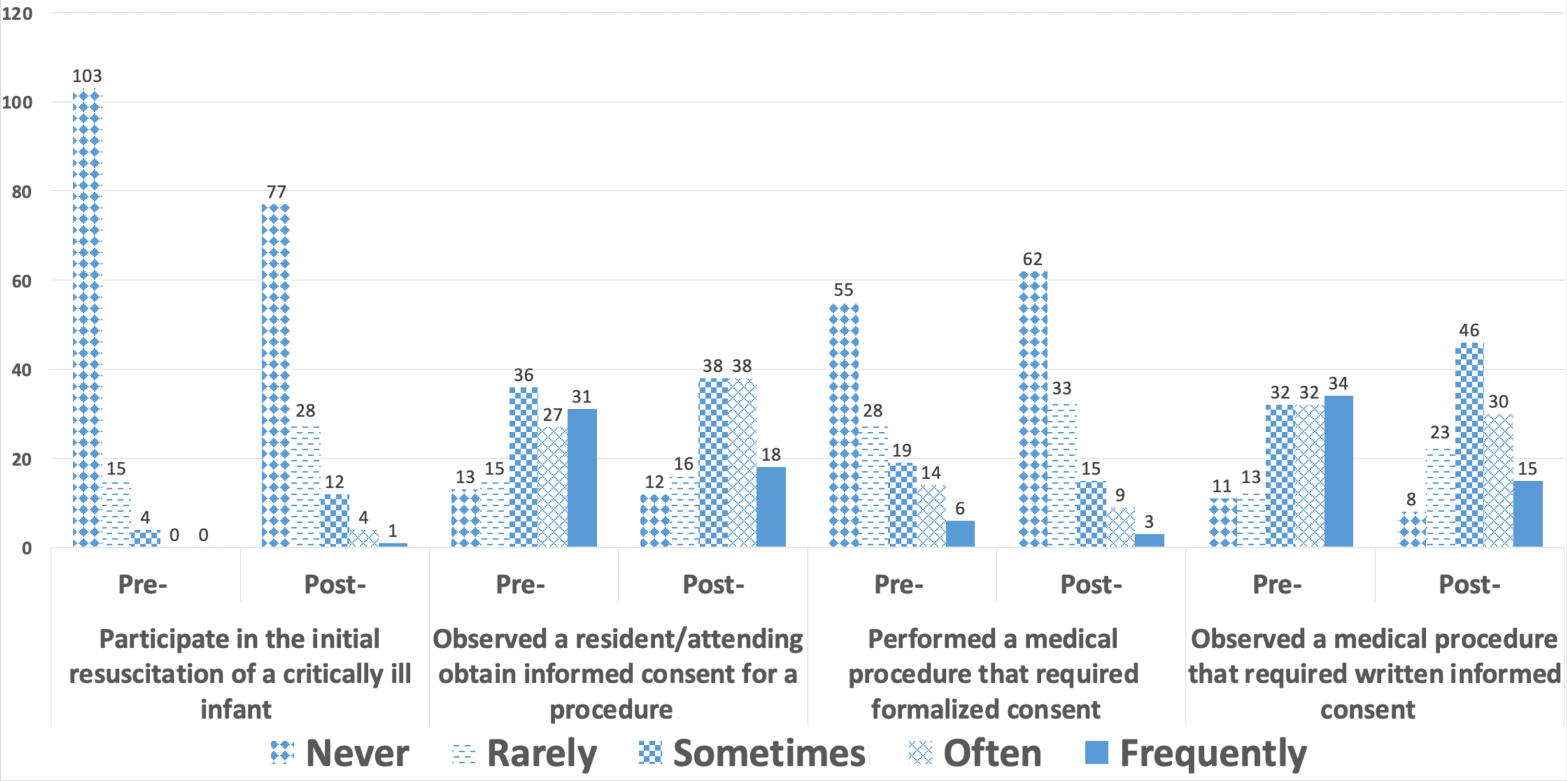
Frequency of selected pediatric clinical activities performed during the pediatrics clerkship Q2 Experiential Questions: During your pediatrics clerkship, how frequently did you perform the following: Responses to experiential question items were reported using a 5-point scale (1=never, 2=rarely, 3=sometimes, 4=often, 5=frequently).

At the conclusion of the clerkship, the study team evaluated students' clinical experiences to determine the impact of the simulation curriculum on these experiences and on students' self-efficacy. We surveyed students using experiential learning questions about the opportunities to engage in specific clinical tasks, including obtaining informed consent, performing procedures, and participating in resuscitation efforts. Experiential learning was rated on a 5-point Likert scale: 1=never, 2=rarely, 3=sometimes, 4=often, and 5=frequently. The results suggest significant improvements in certain areas of experiential learning, particularly in resuscitation efforts. Table 2 presents the comparative analysis of the pre- and post-survey median scores for these measures within the experiential learning domain. Among the 122 respondents who completed the post-curriculum survey, outcomes in the experiential learning domain varied across the four measures assessed (Figure 2). A significant improvement was observed (p<0.01) in students' participation in the initial resuscitation of a critically ill infant, with the median score increasing from 1.19 to 1.54. This slight improvement indicated their participation had increased from "never" to "rarely". In contrast, no significant change was noted in students' observations of a resident or attending physician obtaining informed consent for a procedure (p=0.36), with only minor median score changes, from 3.14 to 3.13, both indicating "sometimes" for this statement. This suggests that the students did not have additional opportunities to observe informed consent procedures. Similarly, there was no significant difference (p=0.08) in the students' involvement in performing a medical procedure requiring formal informed consent, with median scores declining from 2.03 to 1.81. The decrease, although not statistically significant, reflects a slight reduction in participation in this experiential learning area. In addition, a significant decrease was observed in the number of students reporting having observed a medical procedure requiring written informed consent (p<0.01), with median scores decreasing from 3.25 to 3.05, both indicating "sometimes".

**Table 2:** Comparative analysis of pre- and post-survey median scores for experiential learning measures

Question	Average median score of question pre-simulation on a 5-point Likert scale	Average median score of question post-simulation on a 5-point Likert scale	P-value
Experiential Questions: During your pediatrics clerkship, how frequently did you perform the following:			
Participate in the initial resuscitation of a critically ill infant	1.19	1.54	<0.01
Observed a resident/attending obtain informed consent for a procedure	3.14	3.13	0.36
Performed a medical procedure that required formalized consent	2.03	1.81	0.08
Observed a medical procedure that required written informed consent	3.25	3.05	<0.01

**Figure 3:**
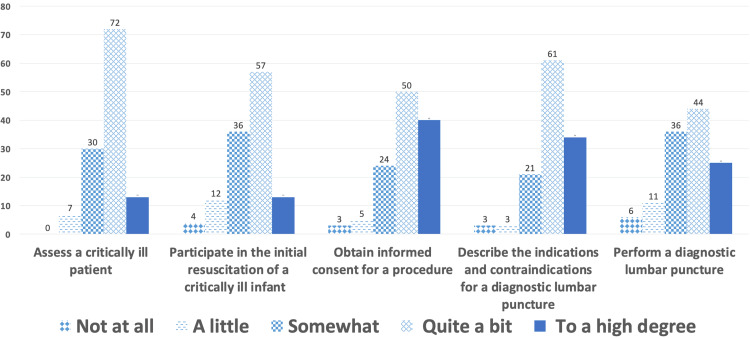
Post-intervention perceptions of the extent to which the combined simulation exercise during the first week of the pediatrics clerkship increased learners' ability to _____. Q3: Experiential Questions (Post-Survey): To what extent do you feel that the combined simulation exercise conducted during your first week of the pediatrics clerkship increased your ability to _______. Responses were reported using a 5-point scale (1=never, 2=rarely, 3=sometimes, 4=often, 5=frequently).

Lastly, in the post-survey, the simulation exercise was evaluated based on how much it helped students engage with key clinical tasks during their clerkship. Questions in this domain specifically sought to explore whether the simulation facilitated their ability to engage in various tasks (Figure 3). The median score results are presented in Table 3 and depict results based on a 5-point Likert scale: 1=not at all, 2=a little, 3=somewhat, 4=quite a bit, and 5=to a high degree. On average, responses across all questions leaned toward "somewhat", demonstrating that the simulation session successfully provided a meaningful foundation for participants to engage with key clinical tasks.

**Table 3:** Comparative analysis of pre- and post-survey median scores for the arithmetic means of median experiential learning measures

Question	Average median score of question on a 5-point Likert scale
Experiential Questions: To what extent do you feel that the combined simulation exercise conducted during your first week of the pediatrics clerkship increased your ability to ______.	
Assess a critically ill patient	3.75
Participate in the initial resuscitation of a critically ill infant	3.52
Describe the indications and contraindications for a diagnostic lumbar puncture	3.98
Perform a diagnostic lumbar puncture	3.58

Cognitive assessment domain

A pre- and post-survey were administered to assess the impact of the simulation curriculum on students' knowledge. The instrument included 10 multiple-choice questions evaluating students' ability to recognize and manage neonatal sepsis, obtain informed consent, and perform a lumbar puncture. The post-simulation assessment at the end of the pediatric clerkship demonstrated marked improvement in students' knowledge of neonatal sepsis management and their understanding of the indications and contraindications of diagnostic lumbar puncture. This is essential for healthcare providers, as a comprehensive understanding of procedures is key to patient safety and management. We observed an increase in overall post-assessment scores (from a pre-test score of 70 to a post-test score of 80 out of 100 points) for all participants, with a significant p<0.01, indicating consistent and substantial improvement in knowledge from the simulation curriculum.

## Discussion

Simulation medicine has changed the landscape of medical education. Students need a safe, non-judgmental environment to learn to perform EPAs before entering clinical settings. Simulation gives students equal exposure to core clerkship objectives they may not encounter in a real clinical setting. Our study showed varied experiential learning outcomes among students, and we observed no significant changes in students' experiences between the pre- and post-simulation curriculum surveys. This may be attributed to variability in clinical encounters during their clerkship, which can impact their self-efficacy or experiential ratings.

Studies have demonstrated that simulation sessions are often the only opportunity students have to engage with these critical scenarios. For example, Brim et al. found that while myocardial infarction pathology and management are core learning objectives in an internal medicine clerkship, only 22% of students encountered this condition outside their simulation sessions [13]. Similarly, studies by Dudas et al. and Friederichs et al. further emphasize the importance of simulation in ensuring comprehensive exposure to essential clinical scenarios [7,14]. Using simulations, students can practice and increase their skill sets without repercussions or patient harm while learning from their mistakes [15-17].

Theories of adult learning emphasize that errors during the learning process can lead to anxiety, fatigue, and educational trauma, decreasing intrinsic motivation and harming education [18]. These challenges can be mitigated by providing a safe learning environment that does not endanger the students or the patients. Furthermore, educators can transform passive learning into active learning using adult learning theories. According to Knowles' principles of adult learning, adult learners tend to learn better when their learning has personal application, when they solve problems, and when education is an active role for them. By giving students in their third year space to manage urgent and emergent triages in simulated contexts, we allow them to bring the knowledge they acquired in the pre-clerkship period to life, facilitating experiential learning. This aligns with the theory of situated cognition, which holds that learning is optimal when it occurs in a realistic clinical context. Importantly, simulated pressure approximates pressures in life while maintaining psychological safety, thereby enhancing skill-building and motivation on the course. The structured debrief facilitates active learning by eliciting reflection, clarifying choice points, and solidifying clinical thinking, crucial building blocks in adult learning, where experience is built to a deeper understanding.

The positive changes observed in pre- and post-survey results suggest that this simulation-based educational approach enhances medical students' self-efficacy. Furthermore, studies by Brim et al. and Friederichs et al. indicate that students prefer interactive sessions to traditional didactics to improve their learning experience and many requested more sessions [7,13]. Some have suggested that the time spent in simulation sessions may have been more valuable and relevant during training, as it provided more opportunities for active involvement in patient care. As both Robertson et al. and Vukin et al. suggested in separate studies, standardized simulation-based education provided relevant and valuable clinical skills, demonstrating higher learning outcomes than traditional preclinical methods alone [17,19]. As such, simulation can provide students with opportunities to engage with clinical scenarios they may not encounter in real settings, fostering deeper engagement and more meaningful learning.

In terms of cognitive assessment, our study shows a significant difference in pre-test and post-test scores before and after the simulation. Alluri et al. reported similar findings in a study comparing lecture-based to simulation-based education among preclinical medical students [20]. Simulation education has also been shown to improve not only preclinical scores but also performance during clinical rotations, as demonstrated in our study. Similarly, Vattanavanit et al. report improvements in medical students' knowledge during septic shock resuscitation during their internal medicine rotation [21]. Beyond improving cognitive knowledge tested by written exams, simulation education has been shown to enhance assessment and management skills. In the study by DeWaay et al., fourth-year medical students demonstrated significant improvement in managing acute coronary syndrome during a clinical practice exam compared with those who relied solely on didactics [22]. Our novel simulation-based curriculum is notable for aligning with AAMC core EPAs for pre-clerkship students, particularly those difficult to observe in clinical settings, to support competency-based learning. Students in this study reported increased confidence in assessing and participating in the initial resuscitation of critically ill patients. This emphasizes the inherent value of simulation-based education in fostering practical clinical skills for emergency scenarios in a controlled, safe environment. Our study findings highlight the beneficial effects of a targeted EPA-based simulation curriculum on our students' confidence, knowledge, and procedural skills while mitigating the ethical risks of granting medical students full autonomy over critically ill patients [15,16,19,23]. This study also highlights the importance of obtaining informed consent for medical procedures. This shows that simulation education extends beyond the acquisition of clinical skills to encompass ethical and logistical training. Effective communication and decision-making are crucial aspects of patient care, and simulation-based education can provide a comprehensive approach to both [17]. In addition to expanding participants' clinical skills, the improvements in their knowledge of neonatal sepsis and lumbar puncture after the simulation underscore the curriculum's value in enhancing core pediatric content. The sustained increase in assessment scores and the improvement in knowledge indicate that simulation-based training can effectively supplement traditional didactic methods. This is likewise supported by Dudas et al., whose study found that, with a simulation-based curriculum, clerkship scores improved compared with those of students who underwent traditional didactics alone [14].

Given the beneficial impact of simulation learning, many clerkship directors reported that they had attempted to integrate more simulation sessions for their students [14]. The findings from this study underscore the importance of experiential learning in medical education. While there was significant improvement in certain areas (such as participation in resuscitation), other areas, such as informed consent and procedural involvement, continued to pose ongoing challenges to ensuring that students gain comprehensive hands-on experience. Given this, our simulation exercise played an invaluable role in clerkship training by providing an opportunity to practice these skills. Our results overall illustrated the impact of simulation in creating a supportive, interactive learning experience that fosters the development of foundational skills essential early in clerkship training. These findings also confirm the need for simulation in the curriculum to help bridge the gap between theoretical knowledge and undergraduate medical training and clinical practice.

Limitations 

While we believe this curriculum is effective, several limitations must be discussed. A significant limitation is the lack of direct observation of student skills within the clinical environment during the clerkship phase. While knowledge was directly measured, the reported improvements in confidence and procedural competency were based on student self-assessment. We are unable to comment on whether these specific skills were performed well during their rotation or if our curriculum directly resulted in real-world improvement at the bedside. Our study demonstrated short-term improvement in knowledge retention and self-efficacy. While the absence of a control group limits our ability to attribute these improvements solely to the curriculum intervention, institutional mandates required providing a uniform educational experience for all clerkship students. This represents an area for potential future work. Further research is needed to assess the long-term retention of skills and knowledge acquired through this EPA-based simulation curriculum. As we only surveyed students at the conclusion of the pediatrics clerkship, it is unclear whether these skills would persist throughout the clerkship and into the transition to residency, where they are more likely to be required. Also, our curriculum did not provide for a direct, final assessment of individual skills competency, a necessary component of true Competency-Based Medical Education. From an implementation perspective, limitations include the availability of clinical faculty to run simulation cases and conduct debriefing sessions; at our institution, faculty volunteered to teach without any specific compensation or time deferral from other responsibilities. The availability of appropriate task trainers with replacement parts could also pose a barrier due to funding constraints. 

## Conclusions

Our study demonstrates the effectiveness of an EPA-based simulation curriculum in enhancing knowledge and self-efficacy in neonatal resuscitation, informed consent, and procedural performance among third-year medical students during the pediatric clerkship. The positive impact on students' reported self-efficacy and procedural competency supports the early integration of simulation-based education into medical curricula. Educators must provide opportunities for medical students to develop confidence and competence in essential clinical skills and ethical decision-making, even when these opportunities may not be available in the clinical learning environment. This helps create a framework under which true competency assessments and entrustment decisions could be developed in the future for these EPAs. Further research is needed to assess long-term retention and the application of these skills in clinical settings. 

## Appendices

Appendix A

**Table 4:** Pediatric clerkship simulation pre-survey

Section	Question #	Question text	Prompt stem	Scale/answer choices
Self-Efficacy Questions	1	Assess a critically ill pediatric patient.	I feel able to _____.	Strongly disagree; Disagree; Neutral; Agree; Strongly agree
Self-Efficacy Questions	2	Participate in the initial resuscitation of a critically ill infant.	I feel able to _____.	Strongly disagree; Disagree; Neutral; Agree; Strongly agree
Self-Efficacy Questions	3	Obtain informed consent for a medical procedure.	I feel able to _____.	Strongly disagree; Disagree; Neutral; Agree; Strongly agree
Self-Efficacy Questions	4	Describe the indications and contraindications of a diagnostic lumbar puncture.	I feel able to _____.	Strongly disagree; Disagree; Neutral; Agree; Strongly agree
Self-Efficacy Questions	5	Perform a diagnostic lumbar puncture.	I feel able to _____.	Strongly disagree; Disagree; Neutral; Agree; Strongly agree
Experiential Questions	6	Participated in the resuscitation of a critically ill infant?	Prior to the pediatrics clerkship, how frequently did you perform the following:	Never; Rarely; Sometimes; Often; Frequently
Experiential Questions	7	Observed a resident/attending obtain informed consent for a procedure?	Prior to the pediatrics clerkship, how frequently did you perform the following:	Never; Rarely; Sometimes; Often; Frequently
Experiential Questions	8	Performed a medical procedure that required written informed consent?	Prior to the pediatrics clerkship, how frequently did you perform the following:	Never; Rarely; Sometimes; Often; Frequently
Experiential Questions	9	Observed a medical procedure that required written informed consent?	Prior to the pediatrics clerkship, how frequently did you perform the following:	Never; Rarely; Sometimes; Often; Frequently
Didactic Questions	10	When performing lumbar puncture in infants, what is the correct anatomical position for needle insertion?	-	L1-L1; L2-L3; L4-L5; L5-S1
Didactic Questions	11	For children under 28 days, what is the recommended prophylactic treatment for presumed meningitis?	-	Ampicillin and Cefotaxime; Vancomycin and Zosyn; Ceftriaxone and Gentamycin; Vancomycin and Penicillin
Didactic Questions	12	Which of the following diagnostic strategies listed below is the most appropriate for a febrile patient under the age of 28 days?	-	CBC with differential, metabolic panel, and blood culture; CBC with differential, metabolic panel, urinalysis, and chest X-ray; CBC with differential, urine culture, and CSF studies; CBC with differential, urine culture, blood culture, and CSF studies
Didactic Questions	13	You are assessing a four-month-old child who is brought to the pediatric emergency department with altered mental status. The child is lethargic, tachypneic without increased work of breathing, and has delayed capillary refill. Supplemental oxygen is applied, and vascular access is obtained. What is the next most appropriate action?	-	Send BMP; if bicarbonate is <15, start 1.5× maintenance IV fluids; Start 1.5× maintenance IV fluids; Administer 20 mL/kg isotonic IV fluid bolus; Administer 20 mL/kg hypotonic IV fluid bolus
Didactic Questions	14	In a patient with full capacity, failure to obtain consent can result in which of the following?	-	No repercussions if the physician deems the procedure medically necessary; The physician may be subject to criminal charges and civil liability; No repercussions if two attending physicians sign off on necessity; No repercussions if consent is obtained from an immediate family member
Didactic Questions	15	True or false: Parents may withhold consent for a life-saving procedure for their child.	-	True; False
Didactic Questions	16	When obtaining informed consent with a non-English patient and/or parents, which of the following is the most appropriate method for interpretation?	-	A family member at bedside; Certified hospital interpreter; Hospital employee who is a native speaker; Google translator
Didactic Questions	17	A five-year-old child presents to the emergency department with altered mental status. The child is ill-appearing. The blood pressure is 60/30 and heart rate is 78. A nurse has tried twice to obtain peripheral vascular access and has been unsuccessful. What is the next best step?	-	Place an ultrasound-guided peripheral IV; Insert a femoral central venous catheter; Have the physician attempt to place a peripheral IV catheter; Insert an intraosseous line
Didactic Questions	18	An eight-month-old male with no past medical history presents to your emergency department with three days of fever (Tmax 39°C) and one day of altered mental status. As per the caregiver, vaccines are not up to date, and there are no sick contacts at home. Physical exam is significant for bilateral enlargement of optic discs on fundoscopic exam. UA and CXR are negative. Which of the following represents the appropriate order of next steps?	-	CT head, lumbar puncture, empiric antibiotics, hospital admission; CT head, empiric antibiotics, hospital admission; Lumbar puncture, empiric antibiotics, hospital admission; Lumbar puncture, defer empiric antibiotics until CSF cell count and protein/glucose, hospital admission
Didactic Questions	19	Which of the following are contraindications associated with performing a lumbar puncture in infants?	-	Increased intracranial pressure; Bleeding diathesis; Evidence of soft tissue infection at the puncture site; Cardiopulmonary instability; All the above

Appendix B

**Table 5:** Pediatric clerkship simulation post-survey

Section	Question #	Question text	Prompt/stem	Answer choices/scale
Self-Efficacy Questions	1	Assess a critically ill pediatric patient.	Please respond to the following prompt: I feel able to _____.	Strongly disagree; Disagree; Neutral; Agree; Strongly agree
Self-Efficacy Questions	2	Participate in the initial resuscitation of a critically ill infant.	Please respond to the following prompt: I feel able to _____.	Strongly disagree; Disagree; Neutral; Agree; Strongly agree
Self-Efficacy Questions	3	Obtain informed consent for a medical procedure.	Please respond to the following prompt: I feel able to _____.	Strongly disagree; Disagree; Neutral; Agree; Strongly agree
Self-Efficacy Questions	4	Describe the indications and contraindications of a diagnostic lumbar puncture.	Please respond to the following prompt: I feel able to _____.	Strongly disagree; Disagree; Neutral; Agree; Strongly agree
Self-Efficacy Questions	5	Perform a diagnostic lumbar puncture.	Please respond to the following prompt: I feel able to _____.	Strongly disagree; Disagree; Neutral; Agree; Strongly agree
Experiential Questions	6	Participated in the resuscitation of a critically ill infant?	During your pediatrics clerkship, how frequently did you perform the following:	Never; Rarely; Sometimes; Often; Frequently
Experiential Questions	7	Observed a resident/attending obtain informed consent for a procedure?	During your pediatrics clerkship, how frequently did you perform the following:	Never; Rarely; Sometimes; Often; Frequently
Experiential Questions	8	Performed a medical procedure that required written informed consent?	During your pediatrics clerkship, how frequently did you perform the following:	Never; Rarely; Sometimes; Often; Frequently
Experiential Questions	9	Observed a medical procedure that required written informed consent?	During your pediatrics clerkship, how frequently did you perform the following:	Never; Rarely; Sometimes; Often; Frequently
Simulation Impact Questions	10	Assess a critically ill patient?	To what extent do you feel that the combined simulation exercise conducted during the pediatrics clerkship increased your ability to ______________	Not at all; A little; Somewhat; Quite a bit; To a high degree
Simulation Impact Questions	11	Participate in the initial resuscitation of a critically ill infant?	To what extent do you feel that the combined simulation exercise conducted during the pediatrics clerkship increased your ability to ______________	Not at all; A little; Somewhat; Quite a bit; To a high degree
Simulation Impact Questions	12	Obtain informed consent for a procedure?	To what extent do you feel that the combined simulation exercise conducted during the pediatrics clerkship increased your ability to ______________	Not at all; A little; Somewhat; Quite a bit; To a high degree
Simulation Impact Questions	13	Describe the indications and contraindications for a diagnostic lumbar puncture?	To what extent do you feel that the combined simulation exercise conducted during the pediatrics clerkship increased your ability to ______________	Not at all; A little; Somewhat; Quite a bit; To a high degree
Simulation Impact Questions	14	Perform a diagnostic lumbar puncture?	To what extent do you feel that the combined simulation exercise conducted during the pediatrics clerkship increased your ability to ______________	Not at all; A little; Somewhat; Quite a bit; To a high degree
Didactic Questions	15	When performing lumbar puncture in infants, what is the correct anatomical position for needle insertion?	-	L1-L1; L2-L3; L4-L5; L5-S1
Didactic Questions	16	For children under 28 days, what is the recommended prophylactic treatment for presumed meningitis?	-	Ampicillin and Cefotaxime; Vancomycin and Zosyn; Ceftriaxone and Gentamycin; Vancomycin and Penicillin
Didactic Questions	17	Which of the following diagnostic strategies listed below is the most appropriate for a febrile patient under the age of 28 days?	-	CBC with differential, metabolic panel, and blood culture; CBC with differential, metabolic panel, urinalysis, and chest X-ray; CBC with differential, urine culture, and CSF studies; CBC with differential, urine culture, blood culture, and CSF studies
Didactic Questions	18	You are assessing a four-month-old child who is brought to the pediatric emergency department with altered mental status. The child is lethargic, tachypneic without increased work of breathing, and has delayed capillary refill. Supplemental oxygen is applied, and vascular access is obtained. What is the next most appropriate action?	-	Send BMP; if bicarbonate is <15, start 1.5× maintenance IV fluids; Start 1.5× maintenance IV fluids; Administer 20 mL/kg isotonic IV fluid bolus; Administer 20 mL/kg hypotonic IV fluid bolus
Didactic Questions	19	In a patient with full capacity, failure to obtain consent can result in which of the following?	-	No repercussions if the physician deems the procedure medically necessary; The physician may be subject to criminal charges and civil liability; No repercussions if two attending physicians sign off on necessity; No repercussions if consent is obtained from an immediate family member
Didactic Questions	20	True or false: Parents may withhold consent for a life-saving procedure for their child.	-	True; False
Didactic Questions	21	When obtaining informed consent with a non-English patient and/or parents, which of the following is the most appropriate method for interpretation?	-	A family member at bedside; Certified hospital interpreter; Hospital employee who is a native speaker; Google translator
Didactic Questions	22	A five-year-old child presents to the emergency department with altered mental status. The child is ill-appearing. The blood pressure is 60/30 and heart rate is 78. A nurse has tried twice to obtain peripheral vascular access and has been unsuccessful. What is the next best step?	-	Place an ultrasound-guided peripheral IV; Insert a femoral central venous catheter; Have the physician attempt to place a peripheral IV catheter; Insert an intraosseous line
Didactic Questions	23	An eight-month-old male with no past medical history presents to your emergency department with three days of fever (Tmax 39°C) and one day of altered mental status. As per the caregiver, vaccines are not up to date, and there are no sick contacts at home. Physical exam is significant for bilateral enlargement of optic discs on fundoscopic exam. UA and CXR are negative. Which of the following represents the appropriate order of next steps?	-	CT head, lumbar puncture, empiric antibiotics, hospital admission; CT head, empiric antibiotics, hospital admission; Lumbar puncture, empiric antibiotics, hospital admission; Lumbar puncture, defer empiric antibiotics until CSF cell count and protein/glucose, hospital admission
Didactic Questions	24	Which of the following are contraindications associated with performing a lumbar puncture in infants?	-	Increased intracranial pressure; Bleeding diathesis; Evidence of soft tissue infection at the puncture site; Cardiopulmonary instability; All the above
Feedback on Activity	25	Overall comments on session	-	Enter your answer
